# Deformation behavior of metallic glasses with shear band like atomic structure: a molecular dynamics study

**DOI:** 10.1038/srep30935

**Published:** 2016-08-02

**Authors:** C. Zhong, H. Zhang, Q. P. Cao, X. D. Wang, D. X. Zhang, U. Ramamurty, J. Z. Jiang

**Affiliations:** 1 International Center for New-Structured Materials (ICNSM), Laboratory of New-Structured Materials, State Key Laboratory of Silicon Materials, and School of Materials Science and Engineering, Zhejiang University, Hangzhou, 310027, People’s Republic of China; 2 Department of Chemical and Materials Engineering, University of Alberta, Edmonton, Alberta, T6G 2V4, Canada; 3 State Key Laboratory of Modern Optical Instrumentation, Zhejiang University, Hangzhou, 310027, People’s Republic of China; 4 Department of Materials Engineering, Indian Institute of Science, Bangalore-560 012, India

## Abstract

Molecular dynamics simulations were employed to investigate the plastic deformation within the shear bands in three different metallic glasses (MGs). To mimic shear bands, MG specimens were first deformed until flow localization occurs, and then the volume of the material within the localized regions was extracted and replicated. Homogeneous deformation that is independent of the size of the specimen was observed in specimens with shear band like structure, even at a temperature that is far below the glass transition temperature. Structural relaxation and rapid cooling were employed to examine the effect of free volume content on the deformation behavior. This was followed by detailed atomic structure analyses, employing the concepts of Voronoi polyhedra and “liquid-like” regions that contain high fraction of sub-atomic size open volumes. Results suggest that the total fraction of atoms in liquid-like regions is a key parameter that controls the plastic deformation in MGs. These are discussed in the context of reported experimental results and possible strategies for synthesizing monolithic amorphous materials that can accommodate large tensile plasticity are suggested.

Plastic deformation in metallic glasses (MGs) at relatively low homologous temperatures (*T*/*T*_*g*_, where *T* is the temperature of deformation and *T*_*g*_ is the glass transition temperature) is characterized by localization of flow into narrow regions within the volume of the material^1^,^2^,^3^. Since these regions, which are often referred as shear bands (SBs), determine many mechanical properties of the MGs, especially their ductility and toughness^2^,^3^, there has been considerable interest in understanding how they form and propagate under applied stress, how to prevent them from unhindered propagation^4^,^5^,^6^, what size effects are induced by shear bands^7^,^8^,^9^,^10^,^11^,^12^,^13^,^14^,^15^,^16^,^17^,^18^, etc. Both experiments^19^,^20^,^21^,^22^,^23^,^24^,^25^,^26^,^27^,^28^ and molecular dynamics (MD) simulations^29^,^30^,^31^,^32^,^33^,^34^,^35^,^36^,^37^,^38^,^39^,^40^,^41^,^42^ were utilized for this purpose. Detailed transmission electron microscopy^19^,^20^,^21^, differential scanning calorimetry^22^, and positron lifetime spectroscopy^23^ experiments have established that SBs in MGs contain higher free volume content, and hence are less dense, as compared to the matrix (MA). The high free volume content also makes them softer than the matrix, which in turn leads to further strain localization or strain softening during plastic flow^22^,^27^,^28^. Their low density enhances the diffusion rates within the shear bands^26^, and makes them susceptible to corrosion^24^,^25^. Very recently, short-range order (SRO) and medium-range order (MRO) atomic structures of Cu_64_Zr_36_ MG based SBs were studied by Feng *et al.*^32^. They found that Zr-centered [0,2,8,5] clusters have a strong tendency to aggregate and build interpenetrating networks with other [0,2,8,5] clusters, demonstrating the planar-like fractals fashion.

While it is well established that plastic flow essentially is confined to a narrow SB once it forms, “what is the constitutive stress-strain behavior of the material within the SB?” is still a question that has not been answered hitherto. Consequent and important questions that arise are “what is the atomic packing structure in the SB?” and “are there any unique structural features associated with the SB?” Since SBs are narrow, i.e., with a few tens of nm thickness^3^, direct experimental examination of their mechanical behavior is rather difficult if not impossible. In this work, we attempt to answer the above-raised questions by the MD simulations of deformation of SBs in three MG alloys (Cu_64_Zr_36_, Cu_36_Zr_64_ and Ni_40_Zr_60_). The shear bands were obtained by plastically deforming the corresponding MG specimens. The extracted SB samples were then subjected to further tensile testing. Systematic investigations on the effects of free volume in the SBs were performed through structural relaxation and by varying the cooling rate. Detailed structural analyses were performed to ascertain the changes during deformation in the SBs. Our results identify key parameters for enhancing the ductility in MGs and in turn give strategies for producing ductile and tough MGs.

## Results and Discussion

**Figure 1:**
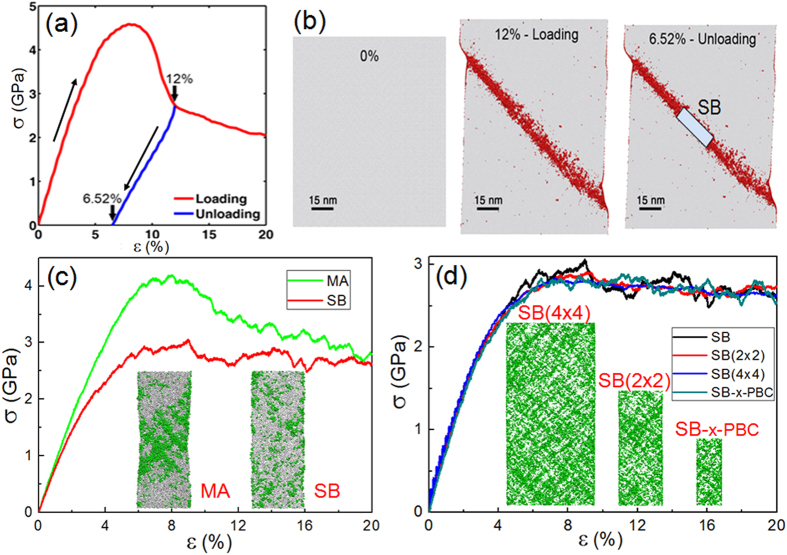
(**a**) Tensile stress-strain (SS) response obtained on the BIG Cu_64_Zr_36_ sample. The unloading line from a strain of 12% is also shown. (**b**) Images showing the distribution of ‘S atoms,’ i.e., atoms that have undergone a von Mises strain of more than 20% (red in color) in undeformed, deformed to 12% tensile strain, and completely unloaded after 12% strain samples. The volume from which the shear band (SB) sample is extracted is shown in the third image with a rectangular box. (**c**) Tensile SS responses of SB and MA samples. Deformation maps at a tensile strain of 20% are shown in the inset. (**d**) Tensile SS curves and deformation maps of SB samples with different sizes.

The tensile stress-strain (SS) response of the MGs with a size of 106.4 (*x*)-117.6 (*y*)-5.6 (*z*) nm^3^ (hereafter named as “BIG sample”) is displayed in Fig. 1(a). After reaching a peak, the stress drops precipitously at a strain of ~12%. This corresponds to the formation of a dominant shear band in the sample, as illustrated in Fig. 1(b). The shear band, which is about 10 nm wide, contains a large number of S-atoms that are colored in red. The BIG sample is subsequently unloaded completely, as shown by the blue line in Fig. 1(a). At zero stress, the plastic strain (or permanent strain) is 6.54%; From the shear banded region of the BIG sample, a sample with ~50,000 atoms and a volume of 8.5 (*x*)-17 (*y*)-5.6 (*z*) nm^3^, which has a high fraction of S-atoms, is cut to make a shear band specimen as shown in Fig. 1(b). This sample is referred as “SB” here afterwards. For comparison purposes, a sample with exactly the same size is also cut from the relatively less deformed ‘matrix’ region, which is referred to as ‘MA’. An identical process is utilized in samples with Cu_36_Zr_64_ and Ni_40_Zr_60_ compositions to generate respective SB and MA samples. For the MD simulations of the latter, the Ni-Zr potential developed by Mendelev *et al.*^43^, was utilized. All the tensile tests were performed at 50 K with a constant strain rate of 1 × 10^8^ s^−1^.

Although we examined three different alloy compositions in this study, most of the results listed below, and the conclusions that are drawn on the basis of them, are similar and appear to be not sensitive to the composition. Therefore, for the sake of brevity, we only list the results obtained on Cu_64_Zr_36_ below and discuss them first. Later, we highlight the results of the other two compositions only in those instances where they diverge significantly from that of Cu_64_Zr_36_.

### Shear band deformation behavior

The tensile stress-strain responses of the SB and MA samples of Cu_64_Zr_36_ are shown in Fig. 1(c). The chemical composition of the SB sample is found to be Cu_64.2_Zr_35.8_, almost the same as the selected MA sample Cu_64.1_Zr_35.9_ within the uncertainty. Note that the homologous temperature of testing is very low (*T*_*g*_ ~940 K^44^ for this alloy). It is apparent from Fig. 1(c) that the mechanical responses of the SB and MA samples differ in a number of different ways, as listed below: (1) Initially, both samples deform elastically with linear stress-strain responses. Fitting to the linear parts yields a Young’s modulus, *E*, of 89.5 GPa for the MA sample, which is similar to that measured on the BIG sample (89.4 GPa). However, it is much higher than *E* of 81.2 GPa obtained for the SB sample. (2) Inelastic deformation in the SB sample starts at a strain of 1.3% whereas it occurs much later at 2.5% in the MA sample. (3) The MA sample shows a peak in stress before softening, just as seen in the BIG sample. The peak stress, σ_*u*_, is about 4.2 GPa, which is slightly less (by about 7%) than 4.5 GPa noted in BIG sample. The stress overshoot behaviors are generally observed in MGs by MD simulations. In recent studies, it was explained by considering the interaction between STZs (shear transformation zones)^45^,^46^ and free volume dynamics^47^. They found that the absolute value of free volume or STZ number (or the liquid-like fraction) is essentially no sense for predicting the homogeneous or localized flow. The amorphous plastic modes is determined by the dynamic activation of STZs and the STZ-mediated free volume dynamics^47^. Further, the stress drop beyond the peak, while it exists, is not as prominent as that seen in the BIG sample. A relatively slow decrease in stress is observed even up to 20% strain, which represents the existence of stress overshoot. In contrast, no prominent peak, i.e., no stress overshoot, can be noted from the stress-strain response of the SB sample. Instead, after nominally continuous nonlinear deformation up to ~6.4% strain, the stress reaches a steady state at about 2.8 GPa, which is about 40% lower than the peak stress noted in the BIG sample. We note that the overshoot in the SS curves was also observed during deformation under pure shear by Cheng *et al.*^38^. They illustrated that the shear stress for initial yielding (τ_over_) reflects the intrinsic resistance to flow initiation of the as-quenched glass, while the stress in the steady-state flow regime (τ_flow_), represents the shear resistance of the rejuvenated glass structure. Here wedid not observe overshoot in the SB samples, i.e. the maximum stress of the SS curves is nearly equal to the flow stress σ_flow_. This observation suggests that the SB sample could have a completely rejuvenated structure^38^. (4) Visualizations of the samples deformed to a strain of 20%, displayed in the inset of Fig. 1(c), show that the deformation in the SB sample is homogeneous with uniform elongation. The atoms with Von Mises strain higher than 0.2 (hereafter named as “S-atoms”) are shown in green. When the SB sample is further tensiled to a strain of 80%, it still exhibits uniform deformation, as shown in Fig. S1. The strain rate effect, if any, was also examined. We did not observe any significant differences for the strain rates of 1 × 10^7^ s^−1^, 1 × 10^8^ s^−1^ and 1 × 10^9^ s^−1^ and the SB samples exhibit homogeneous deformation under studied strain rates. Here we define the “homogeneous deformation” as “uniform elongation with no necking even to a strain as high as 50%”. Necking behaviors are also regarded as one kind of strain localization in this work. Furthermore, the strength of the SB sample was found to be insensitive to the strain rate, as shown in Fig. S2. In addition, it is generally accepted that metallic glasses deformed at higher strain rate incline to cause a localized deformation behavior, i.e., under higher strain rates, then MGs should easily deform in localized mode at lower strain rates. Strain rates of 10^7^–10^9^ s^−1^ used in this work are higher than normal experiments. Thus, when SB samples studied here show homogeneous deformation behavior at high strain rates of 10^7^–10^9^ s^−1^, it is not unreasonable to expect the same deformation mode for lower strain rates could occur in experiments. Of course, more studies in experiments are still needed to clarify this issue. The MA sample, in contrast, exhibits non-uniform or heterogeneous plasticity with perceptible necking, which is similar to that reported in ref. 48. But, in Ni_40_Zr_60_, the relatively rapid-quenched notch-free samples also show uniform elongation and extremely large plasticity before necking happens^48^. Different deformation modes are also achieved by Shi *et al.* where the ratio of deformation participated atoms is also calculated to distinguish the different deformation mode^36^.

Numerous experimental and computational studies reported a change in the deformation mode, i.e., from heterogeneous to homogeneous and in turn enhanced ductility in MGs with extremely small sample dimensions^7^,^8^,^9^,^10^,^11^,^12^,^13^,^14^,^15^,^16^,^17^,^18^. Therefore, the question ‘whether observed homogeneous deformation in current SB sample is a consequence of its small size?’ arises as the thickness of SB is only 8.5 nm. To answer this question, we performed the following simulations. First, two larger specimens with the sizes of 17 (*x*)-34 (*y*)-5.6 (*z*) nm^3^ and 34 (*x*)-68 (*y*)-5.6(*z*) nm^3^, were generated by replicating the SB sample by 2 × 2 and 4 × 4 times in both *x* and *y* directions whereas the thickness of the sample (*z* dimension) was kept constant. After relaxing the samples for 100 ps at 50 K, they were tensile tested with the same condition for testing SB sample. In addition, an SB sample with three-dimensional periodic boundary conditions (PBC), representing a bulk sample, was also tested. The stress-strain responses, displayed in Fig. 1(d), show that the mechanical response of all four specimens are nearly-identical, i.e., no stress overshoot, and all the fours samples exhibit homogeneous plastic deformation even at a strain of 20%. These results conclusively show that the unique response observed in the SB sample in Fig. 1(d) is not a consequence of its small size^7^,^8^,^9^,^10^,^11^,^12^,^13^,^14^,^15^,^16^,^17^,^18^, and is indeed intrinsic to its nature. In the following sections, we examine the possible mechanistic origins for this behavior.

### Effects of structural relaxation on deformation

It is reasonable to expect that the unique deformation behavior observed in the SB sample is a consequence of its specific atomic structure. It is generally believed that the SB regions in plastically deformed MGs are lower in density and hence contain higher free volume than its bulk counterpart^19^,^20^,^21^,^22^,^23^,^24^,^25^,^26^,^27^,^28^,^29^,^30^,^31^,^32^. This naturally leads to the following question: “is the observed homogeneous deformation in the SB sample due to the increased free volume?” It is well known that structural relaxation, which is achieved by annealing the MG below its *T*_*g*_, can reduce the free volume in MG^22^,^44^. Based on this idea, we relaxed the SB sample at six annealing temperatures, *T*_*a*_ (800, 600, 500, 400, 300 and 200 K), all of which are well below *T*_*g*_ and *T* = 1200 K, which is above *T*_*g*_. In all cases, the samples are heated rapidly with a heating rate of 10^13^ K/s from 50 K until *T*_a_ is reached, held at *T*_*a*_ for 5 ns, and then cooled to 50 K with a relatively slower cooling rate of 10^11^ K/s.

**Figure 2:**
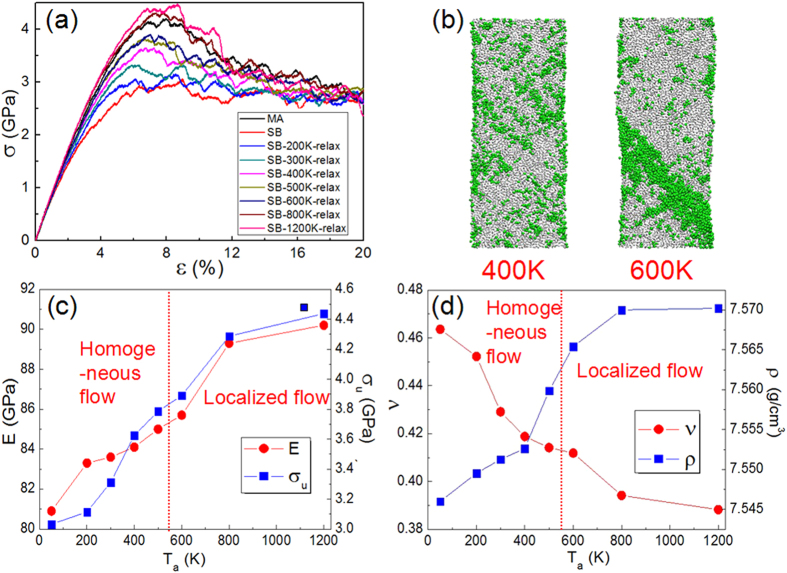
(**a**) Tensile SS curves of Cu_64_Zr_36_ SB samples after annealing at different temperatures. SS curves obtained on SB and MA samples are also shown for comparison. (**b**) Deformation maps (total strain = 20%) of samples annealed at 400 and 600 K, showing localization of flow in the latter. (**c**) Variations of Young’s modulus, *E*, and peak stress, σ_u_, of SB sample with the annealing temperature, *T*_a_. (**d**) Variations of Poisson’s ratio, *v*, and density, *ρ*, with *T*_a_. Dashed lines in (**c**,**d**) indicate approximate *T*_a_ at which homogeneous to localized flow transition occurs.

Tensile stress-strain responses of annealed SB samples are displayed in Fig. 2(a), where the stress-strain curves obtained on the SB and the MA samples are also plotted for comparison. The figure shows with increasing *T*_*a*_, the onset strain of yielding and σ_*u*_ of the relaxed-SB samples increase. Variations of *E* and σ_*u*_ as a function of *T*_*a*_are plotted in Fig. 2(c). A continuous rise in both properties is observed, with the properties of the 800 K annealed sample being similar to that of MA sample, indicating a fully relaxed structure upon annealing at 800 K. Furthermore, annealing at 1200 K results in a material that is stronger than the MA sample, which is consistent with a recent study that optimum annealing is equivalent to a slowly cooled MG in terms of free volume^49^. Deformation maps of the samples that were annealed at 400 and 600 K to a total strain of 20% are displayed in Fig. 2(b). While plasticity in the sample annealed at 600 K indicates localization of flow into a shear band, the deformation is still homogeneous in the sample relaxed at 400 K. While the SB samples annealed at 800 and 1200 K exhibit similar localized flow as was observed in the 600 K sample, samples annealed between 200 and 500 K are similar to the 400 K annealed sample. Variations of the Poisson’s ratio, *v*, and density, *ρ*, of the SB sample after annealing at different *T*_*a*_are shown in Fig. 2(d). Here, *v* was estimated as the ratio of transverse-to-longitudinal strain during elastic deformation of the sample, i.e., within the strain of 4%. It is seen that *v* decreases with increasing *T*_*a*_. The variation of *v* with *T*_*a*_clearly shows that structural relaxation densifies the SB sample, up to a temperature of 800 K. These results clearly show that free volume annihilation is due to structural relaxation, which in turn leads to enhanced *E* and σ_*u*_^19^,^20^,^21^,^22^,^23^,^24^,^25^,^26^,^27^,^28^,^29^,^30^,^31^. Conversely, they suggest that the high free volume content in the SB sample is *raison d'être* for its softness and homogeneous plasticity.

In this context, we note that in the *in-situ* tensile deformation experiments conducted by Schroers *et al.*^18^ on directly molded Pt-based MG nanowires (NWs), it was shown that the ductility could be tunable. While the as-molded NWs are brittle, ion irradiation with Ga^+^, which leads to a distinct glassy state, imparts tensile ductility and quasi-homogeneous plastic flow in the NWs. This behavior could be reversible and the glass returns to a brittle state upon subsequent annealing. These reported results further support our results, i.e., the irradiation process is usually considered as introducing “free volume” in MGs while the annealing process is usually considered as reducing “free volume”. As a result, the fractions of atoms in liquid-like regions could be tunable, resulting in the deformation mode transition.

### Effects of cooling rate

Evidently, the homogeneous deformation in SB can be altered to non-uniform localized plasticity through annealing at high temperatures. However, annealing can reduce the free volume content in a MG sample, and hence cannot be utilized to increase free volume. One possible way to enhance the free volume content in a given metallic glass is to cool the liquid at higher cooling rates. To examine this possibility, the MA sample, which was originally cooled at 10^11^ K/s, was first rapidly heated to 2000 K and relaxed for 1 ns, then quenched at the following rates: *ψ* = 10^12^, 10^13^, 5 × 10^13^, 10^14^, and 10^15^ K/s.

**Figure 3:**
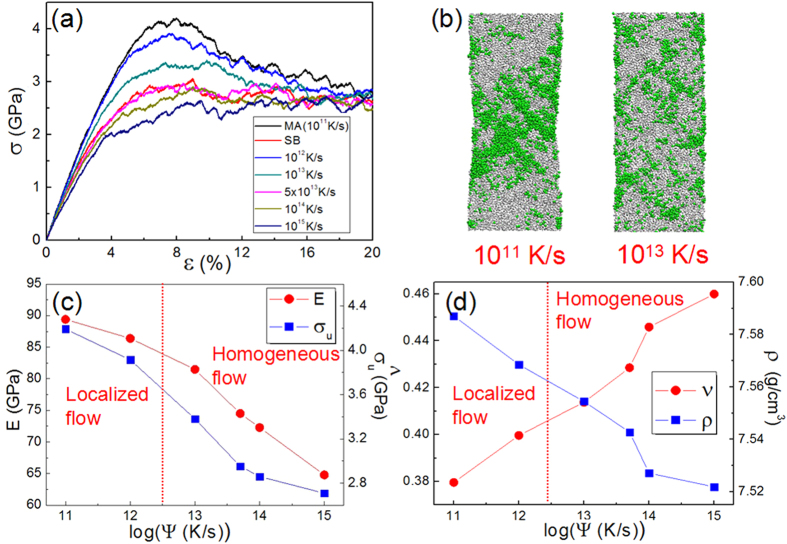
(**a**) Tensile SS curves of Cu_64_Zr_36_ samples which were obtained with different cooling rates, *ψ* (see text for details). SS curves obtained on MA and SB samples are also shown for comparison. (**b**) Deformation maps (total strain = 20%) of MA samples subjected to *ψ* = 10^11^ and 10^13^ K/s; flow localizes in the former whereas it is homogeneous in the latter. (**c**) Variations of Young’s modulus, E, and peak stress, σ_u_, of samples with the cooling rate, *ψ*. (**d**) Variation of Poisson’s ratio, *v*, and density, *ρ*, with *ψ*. Dashed lines in (**c**,**d**) indicate the approximate value at which homogeneous to localized flow transition occurs.

The tensile stress-strain responses of samples with different cooling rates are displayed in Fig. 3(a) along with those of the MA and the SB samples. It is noted that decreasing *ψ* has essentially the same effect as enhanced structural relaxation; both the processes lead to increase σ_*u*_ of MG. Deformation maps of the samples cooled at 10^11^ and 10^13^ K/s are compared in Fig. 3(b). Localization of plastic flow in the sample that was cooled at 10^11^ K/s can be noted whereas the sample cooled at 10^13^ K/s exhibits homogeneous deformation, which is similar to that observed in the SB sample. As shown in Fig. 3(c), a higher *ψ* corresponds to lower *E* and σ_*u*_, since the structure and properties of samples subjected to high *ψ* are more close to SB. It is interesting to note that the samples with high *ψ* such as 10^14^ or 10^15^ K/s show even lower *E* and σ_*u*_ than those of SB. In Fig. 3(d), variations of *v* and *ρ* with *ψ* are displayed, which show that a higher *ψ* results in a MG with higher *v* and lower *ρ*. Similarly, *ρ* in samples with *ψ* = 10^14^ and 10^15^ K/s are even lower than *ρ* of the SB sample, suggesting higher free volume contents in the former.

### Structural analysis

To understand the origin of differences in the mechanical responses, if any, of the annealed SB samples and MA samples cooled at different *ψ*, their local short-range structures were analyzed by Voronoi tessellation.

**Figure 4:**
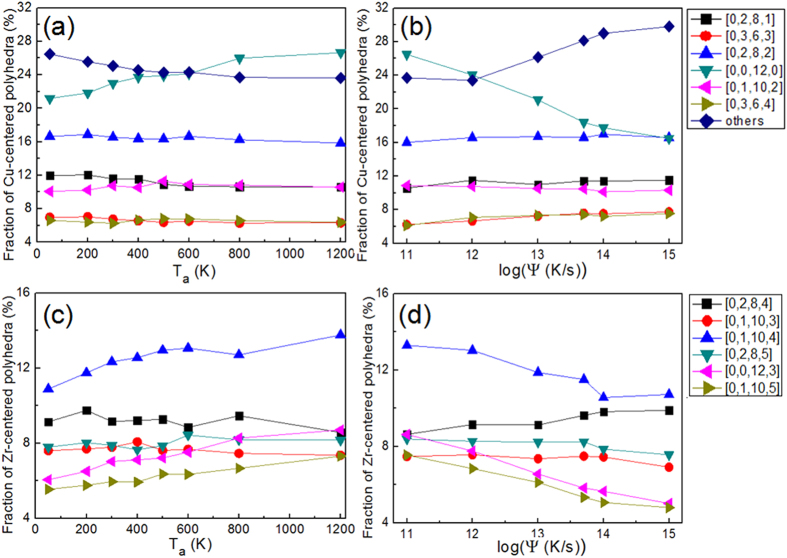
The fractions of Cu-centered Voronoi polyhedra (VP) in the Cu_64_Zr_36_ SB samples (**a**) with the annealing temperature, *T*_a_, and (**b**) with cooling rate, *ψ.* Zr-centered VP in the Cu_64_Zr_36_ SB samples (**c**) with the annealing temperature, *T*_a_, and (**d**) with cooling rate, *ψ.* Only six most abundant VPs are considered.

Figure 4(a) shows six most abundant Cu-centered Voronoi polyhedra (VPs) in samples annealed at different *T*_*a*_. These six VPs constitute more than 70% of the total polyhedra in the sample. From Fig. 4(a), it is seen that in all the annealed SB samples, the full icosahedra (FI) with the indices of [0,0,12,0] is the largest constituent about 24%. Importantly, its fraction gradually increases with increasing *T*_*a*_, whereas the fractions of the other five polyhedra, [0,2,8,1], [0,2,8,2], [0,3,6,3], [0,1,10,2] and [0,3,6,4], remain almost invariant. This observation implies that there is a complementary decrease of the fraction of the other ‘fragmented’ polyhedra (FP) with *T*_*a*_. Thus, the increase in *ρ* with *T*_a_ that is seen in Fig. 3(d) could be linked to the increased FI content. Figure 4(b) shows the fractions of the same VP in samples subjected to different *ψ*. It appears that among them, the fraction of FI is also the highest at all *ψ*. Like what was observed in Fig. 4(a), its decrease with the increase in *ψ* is essentially compensated by an increase in the content of FP. Fractions of the top six Zr-centered VPs in SB are plotted in Fig. 4(c,d) as functions of *T*_*a*_ and *ψ*, respectively. Compared with the Cu-centered VP, the fractions of Zr-centered VP in Cu_64_Zr_36_ are relatively irregular and randomly distributed. The [0,1,10,4] VP, which constitutes the highest fraction, is only 13%, and the total of the six highest Zr-centered VP is less than 50%. As shown in Fig. 4(c), the contents of [0,0,12,3], [0,1,10,4] and [0,1,10,5] VPs, which have high five-fold symmetry order, increase with increasing *T*_*a*_ whereas the contents of other VPs, i.e., [0,2,8,4], [0,1,10,3] and [0,2,8,5], remain nearly constant. Noticeably, these three kinds of Zr-centered VP have high five-symmetry order and also reported to incline to connect with Cu-centered FI forming medium-range order (MRO) in space^50^,^51^,^52^. Thus, the contents of these three Zr-centered VPs are shown the same trend with Cu-centered FI. The SRO and MRO atomic structures in Cu_64_Zr_36_ MG based SBs were recently studied by Feng *et al.*^32^. They found that the fraction of Zr-centered [0,2,8,5] VP was detected to be 7.5% in SB, slight larger than 6.5% in MA, which are consistent with the results for both MA and SB close to 8% obtained here. The MRO is also examined here by the connectivity of the network formed by the central atoms of [0,2,8,5] VP, confirming that Zr-centered [0,2,8,5] VPs have a strong tendency to aggregate and build interpenetrating networks with other [0,2,8,5] VP. However, as Zr-centered [0,2,8,5] VP is only 2.5% contented in all atoms, the effect of these Zr-centered [0,2,8,5] VP networks on properties of the whole sample still needs more studies. At different *ψ*, the fractions of Zr-centered VPs also change, as seen in Fig. 4(d). Similar to the Cu-centered FI, the contents of [0,0,12,3], [0,1,10,4] and [0,1,10,5] also decrease with increasing *ψ* whereas the contents of [0,2,8,4], [0,1,10,3] and [0,2,8,5] remain constant. The population change of VPs in Cu_64_Zr_36_ supports the hypothesis of a network of FI-backbone^51^,^52^, which gets disrupted due to severe plastic deformation that occurs in the SB. Our results show that the backbone can be somewhat recovered by annealing at higher temperature, making the structure and properties of annealed SB closer to those of MA. Hence, a clear correlation between the deformation mode and the underlying atomic structure could be established. In addition, our results imply that the content of Cu-centered FI in the MG plays a key role in affecting the mechanical behavior in Cu_64_Zr_36_ MGs.

### Effect of composition

The results obtained for the Cu_64_Zr_36_ MG confirm that plastic deformation behavior in the shear band-like structure is closely related to the content of Cu-centered FI. It has been reported that the fraction of FI is sensitive to the composition^53^, i.e., FI polyhedra are abundant in Cu-rich alloys such as Cu_64_Zr_36_, while they are low in Cu-poor (or Zr-rich) alloys. Thus, we next examine whether these results on the deformation mode change obtained for the Cu_64_Zr_36_ MG exists also in a Zr-rich Cu-Zr MG. For this purpose, we have selected Cu_36_Zr_64_ and performed both annealing (at *T*_*a*_ = 800, 600, 500, 400, 300, and 200 K) and synthesized specimens with varying *ψ*. The homogeneous to heterogeneous deformation mode transition was indeed found to be at *T*_*a*_ = 500 K or when *ψ* = 10^11^ K/s. All results for the Cu_36_Zr_64_ MG, which are similar to those in Cu_64_Zr_36_, are given in the supplementary material (SM). The strength of the annealed samples increases with *T*_*a*_ and decreases with *ψ*. While the trends in *v* are similar to that seen in Cu_64_Zr_36_, we observe that *ρ* of Cu_36_Zr_64_ is not affected by either *T*_*a*_ or *ψ*. The difference in *ρ* for MA and SB Cu_36_Zr_64_ samples is detected to be only 0.17%, which is much lower than 0.54% observed in Cu_64_Zr_36_ samples. It is not always true that atom packing in shear bands is looser than outside, as reported in a recent experimental work^54^.

**Figure 5:**
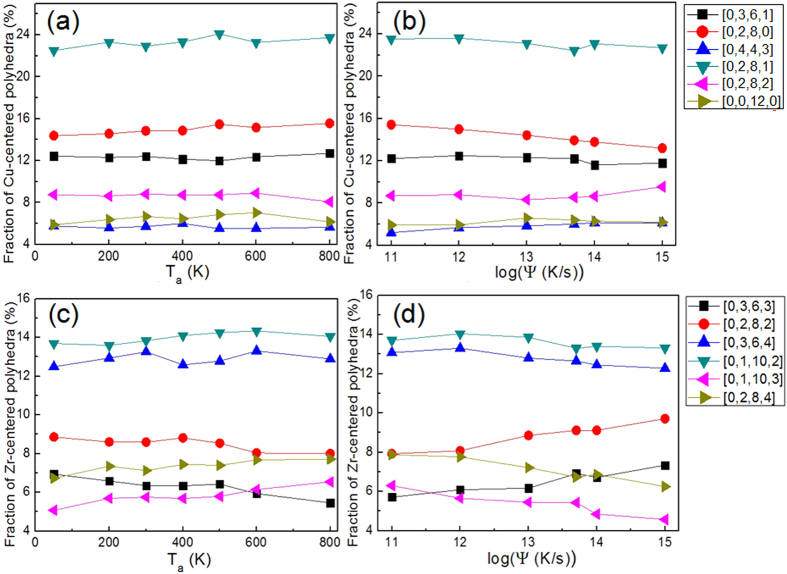
The fractions of Cu-centered Voronoi polyhedra (VP) in the Cu_36_Zr_64_ SB samples (**a**) with the annealing temperature, *T*_a_, and (**b**) with cooling rate, *ψ.* Zr-centered VPs in the Cu_36_Zr_64_ SB samples (**c**) with the annealing temperature, *T*_a_, and (**d**) with cooling rate, *ψ.* Only six most abundant VPs are considered.

Figure 5(a) shows the various fractions of six highest Cu-centered VPs in SB-annealed Cu_36_Zr_64_ samples with different annealing temperatures. Unlike in Cu_64_Zr_36_, the fraction of Cu-centered FI in Cu_36_Zr_64_ is only 5% and VPs with relatively lower CN, such as [0,2,8,1], [0,2,8,0], [0,3,6,1], constitute larger contents in the alloy. Moreover, the fraction of each VP does not vary with *T*_*a*_, despite the fact that mechanical properties do indeed exhibit variation with *T*_*a*_. Figure 5(b) shows the fractions of Cu-centered VPs in samples with different *ψ* in Cu_36_Zr_64_. Similar to what seen in Fig. 5(a), the fractions of Cu-centered FI are less contented while the low-CN VPs have higher fractions and the fraction of each VP remains nearly unchanged. The fractions of Zr-centered VPs, which are dominant in Zr-rich Cu_36_Zr_64_ alloy, remain invariant with *T*_*a*_, as shown in Fig. 5(c). The low-CN VPs such as [0,1,10,2], [0,3,6,4] and [0,2,8,2] are abundant as compared to high-CN VPs such as [0,0,12,3], [0,1,10,4] and [0,1,10,5], which are more in Cu_64_Zr_36_. In Fig. 5(d), the fractions of various VPs as a function of *ψ* are shown. The contents of six major Zr-centered VPs remain almost unchanged and low-CN VPs are again dominant. Consequently, it can be concluded that in Cu_36_Zr_64_ alloy, the homogeneous to heterogeneous deformation mode transition is also detected by varying *T*_*a*_ or *ψ*, although the topological short-range structures are not sensitive to either *T*_*a*_ or *ψ*.

In addition to the binary Cu-Zr systems, we also examined the ‘shear band’ like structure’s influence on mechanical behavior of Ni_40_Zr_60_ MG, for which an empirical potential was reported^43^. After producing SB samples in this system using the processes that are identical to those used in Cu-Zr alloys, they were annealed at 800, 600, 500, 400, 300, and 200 K. The tensile stress-strain responses (shown in SI) show an increase in σ_*u*_ with increasing *T*_*a*_ and decreasing *ψ*, but with no significant change in the mode of deformation, i.e., deformation remains homogeneous for the entire range of *T*_*a*_ and *ψ* investigated in this work. This may be related to the fact that the sample thickness of this study is below the critical thickness required for localized deformation in Ni_40_Zr_60_^7^,^8^,^9^,^10^,^11^,^12^,^13^,^14^,^15^,^16^,^17^,^18^. Notably, the variation in *ρ* in Ni_40_Zr_60_ is similar to what we observed in Cu_36_Zr_64_, with *ρ* of MA sample higher by only 0.07% than that of SB sample. As Ni and Cu are close to each other in the periodic table and the composition of Ni_40_Zr_60_ is also close to Cu_36_Zr_64,_ the types and fractions of Ni-centered VPs in Ni_40_Zr_60_ are similar to those of the Cu-centered VP in Cu_36_Zr_64,_ i.e., the low-CN VP such as [0,2,8,1] has higher fraction and the fraction of each VPs does not vary in any significant manner with different *T*_*a*_ or *ψ*. Some small differences do exist. Lower CN VP such as [0,3,6,1] and [0,2,8,0] that are abundant in Cu_36_Zr_64_ are relatively less in Ni_40_Zr_60_, replaced by several VPs such as [0,2,8,2] or [0,3,6,3] that have a higher CN of 12. The fractions of six highest Zr-centered VP remain invariant with *T*_*a*_ and *ψ* (see SI), having the [0,2,8,4] VP being the most dominant in Ni_40_Zr_60_, in contrast to those seen in both Cu_64_Zr_36_ and Cu_36_Zr_64_. This suggests some fundamental structural differences between Ni-Zr and Cu-Zr systems, and requires further detailed studies. On the basis of the current observations, we conclude that in Ni_40_Zr_60_ the topological short-range structure is nearly identical in each sample and does not change much with either *T*_*a*_ or *ψ*.

### Mechanism of homogeneous flow

The observation of homogeneous deformation in SB samples that were either annealed at relatively low *T*_*a*_ or subjected to high *ψ* in all the three alloys examined in this work suggests that it might be a general, if not universal, feature in MGs. Two different deformation modes must be related to the intrinsic structure of the samples. However, only from the perspectives of *ρ* or the types and numbers of VP present, it is still difficult to determine what these intrinsic factors are. It warrants more structural analyses. We recognize that an MG’s structure is spatially heterogeneous in nature and can be partitioned into solid-like and liquid-like regions depending on the local packing^55^. In the dense random packing hard sphere model, the spheres that closely pack together form solid-like regions while the volumes with disconnected cavities make up of the void regions. Using the algorithm proposed by Sastry^56^, the void regions in a MG can be evaluated by setting suitable radius of each atom, *R*_*a*_, together with an exclusion radius, *R*_*e*_, that will exclude regular tetrahedrons or half-octahedrons that are common even in close-packed crystal structures.

**Figure 6:**
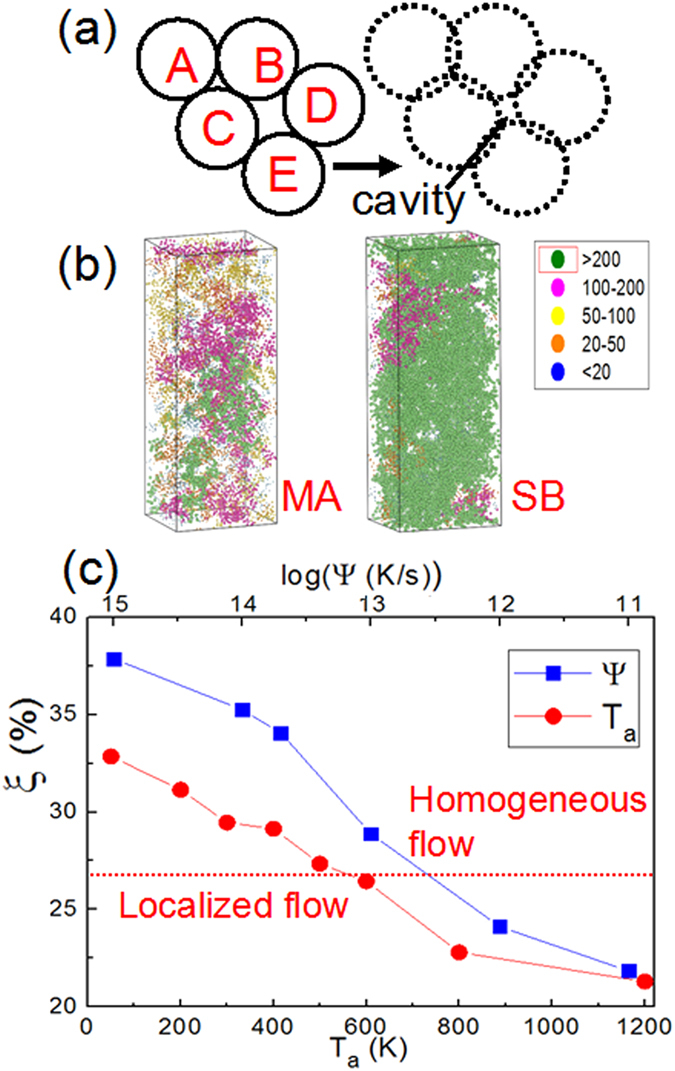
(**a**) Schematic illustration of cavity in an amorphous packing of atoms. (**b**) Distribution of atoms near the cavity regions in MA and SB samples of Cu_64_Zr_36_, forming different sized clusters, which are indicated with different color schemes. (**c**) The fractions of atoms at the near-cavity region, ξ, in the SB sample with the annealing temperature, *T*_a_, and in samples cooled with different rates, *ψ*. The horizontal dashed line indicates the approximate value of 27% at which homogeneous to localized flow transition occurs.

A schematic representation of the cavity in two-dimensional space is shown in Fig. 6(a). The atoms A, B and C are close-packed to form a small cavity in between them, while the atoms B, C, D, and E are relatively loosely packed and hence form a large cavity. By setting proper *R*_*e*_, which is equivalent to the enlarged radius of atoms, for all atoms, the small cavity within the ABC atomic cluster disappears while the cavity within BCDE cluster remains. This process enables us to partition all the atoms in the material into two groups by determining whether or not they are near a cavity^57^,^58^. Figure 6(b) shows the atomic configurations in near-cavity regions in the MA and the SB samples of Cu_64_Zr_36_, where *R*_*e*_ are set as 1.278 and 1.5895 Å for Cu and Zr atoms, respectively^59^. The near-cavity atoms can be connected to form clusters with different sizes, which are shown by different color schemes in Fig. 6(b). It is clear that the fraction of the near-cavity atoms, *ξ*, is very large in the SB sample, forming even larger clusters, as compared to those in MA. *ξ* is as high as 33% in the SB sample, which deforms homogeneously. With annealing, *ξ* reduces with *T*_*a*_, as shown in Fig. 6(c), to reach a value of 21% in 800 K annealed sample, in which plastic flow is localized. Apparently, a higher *ξ* favors homogeneous deformation. Figure 6(c) also shows variation of *ξ* with *ψ*, i.e., *ξ* increases from 22% for *ψ* = 10^11^ K/s to more than 38% for *ψ* = 10^15^ K/s. Since a higher *ξ* also corresponds to homogeneous deformation in the samples with different cooling rates, in a given MG *ξ* determines its mechanical properties and deformation mode, which is independent of the processing history of the MG. The critical value *ξ* for the deformation mode change is close to about 25%, which is estaimted to participate in inelastic deformation made in ref. 60. Based on the percolation theory^61^, the threshold for random close-packed sphere is approximately 0.27. Surprisingly, this number is close to value we observed for the deformation transition between homogeneous flow and localized flow. This suggests the near-cavity atoms percolates the MG sample and form a soft network structure, which, in turn, can accommodate strain while maintaining overall structural integrity of MG during deformation. On the other hand, if *ξ* is smaller than the threshold, stress concentration is more likely to occur in the solid-like region, leading to localized deformation.

**Figure 7:**
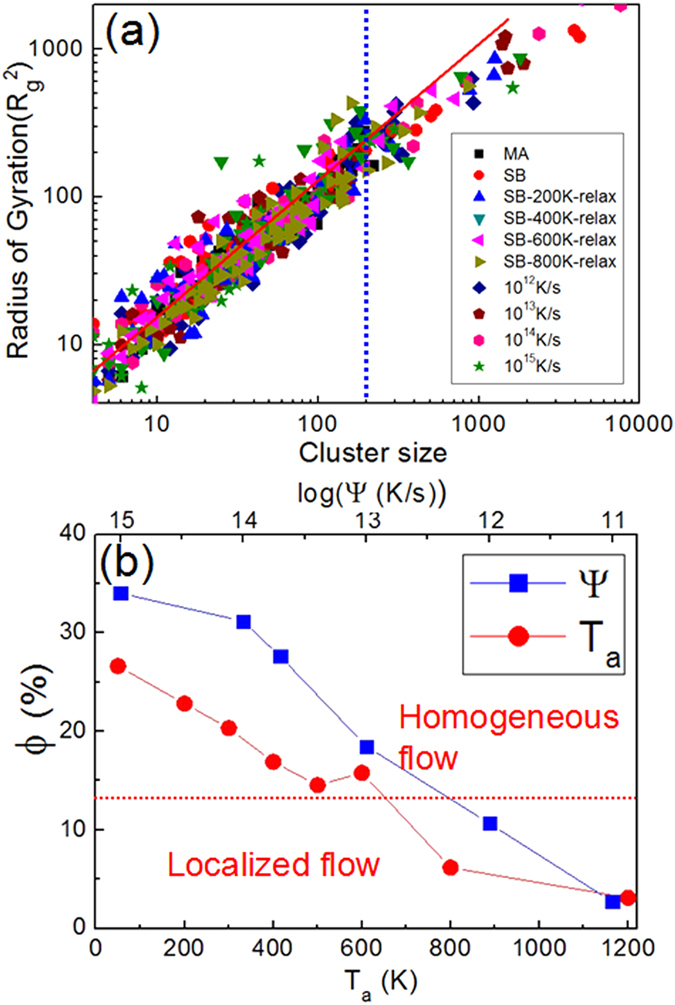
(**a**) Variation of the radius of gyration (*R*_*g*_^2^) with the cluster size in various samples of Cu_64_Zr_36_. A linear scaling is seen for clusters up to ~200, which is indicated by a dashed line. (**b**) Fractions of atoms at liquid-like region, *ϕ*, of SB samples with annealing temperatures, *T*_a,_ and samples subjected to different cooling rates, *ψ*.

The size and shape distributions of the clusters formed by near-cavity atoms are shown in Fig. 7(a). The radius of Gyration (*R*_*g*_^2^) is plotted as a function of the cluster size, *ß*, in various samples of Cu_64_Zr_36_, where 
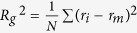
. Here, *r*_*i*_ is the position of each atom in the cluster, *r*_*m*_ is the location of center of mass of clusters and *N* is the number of atoms in clusters. Figure 7(a) indicates that *R*_*g*_^2^ and *ß* are power law related for *ß* ≤ 200 atoms approximately, but deviates from the relationship at higher *ß*. It is possible for the larger-size clusters to percolate, leading to the atoms in them accommodating shear more easily and cooperatively. Thus, the volumes where *ß* is larger than 200 are identified as ‘liquid-like regions’, likely to contribute relatively more to the plastic deformation. Variations of the fraction of atoms in liquid-like regions, *ϕ*, with *T*_*a*_ and *ψ* are shown in Fig. 7(b). As expected, *ϕ* reduces with increasing *T*_*a*_ and decreasing *ψ*. Without doubt, a higher *ϕ* is conducive for strain accommodation by short-range atomic movement, which eventually results in homogeneous deformation. However, if *ϕ* is too small, such local atomic movement is not sufficient enough for the relaxation of the imposed stress throughout the body of the MG, which necessitates flow localization. Variations of *ξ* and *ϕ* for different *T*_*a*_ and *ψ* in Cu_36_Zr_64_ and Ni_40_Zr_60_ are shown in Fig. S6, the trends are similar to those observed as in Cu_64_Zr_36_. It is important to note that the “liquid-like” atoms here are not equal to the GUM (geometrically unfavoured motifs) atoms defined by Ma *et al.*^39^,^40^. However, GUM atoms usually have higher probability of occurrence near sub-atomic sized cavities when compared to the close-packed atoms with high symmetry such as full icosahedra (FI).

## Discussion

Results obtained from present MD simulations suggest that MGs with shear band like structural features, i.e., high uniformly distributed free volume content, could have homogeneous deformation without exhibiting any localization. The deformation behavior is quite similar to the performance in heterogeneously randomized STZ model^62^ and uniaxial compression creep experiments performed on micro-/nano-sized pillars at room temperature^63^. These results suggest that the method based on cavity analysis is a robust way to identify the structure of metallic glasses, as compared with the method based on the evaluation of VP. A higher fraction of near-cavity atoms or liquid-like regions favor homogeneous plasticity while lowering the strength. Indirect experimental evidences supporting the conclusion drawn here were reported in recent published works. Jiang *et al.*^64^ examined the tensile response of 50 nm thick films of Ni_60_Nb_40_ metallic glass, synthesized by magnetron-sputtered method, through *in-situ* tensile measurements in transmission electron microscopy. They found that the ‘as-deposited’ films undergo a total and uniform strain of ~32.6%, before fracture. Putting the two ends of a fractured specimen together, the plastic strain component was estimated as 25.9%, whereas the rest of ~6.7% is elastic. After annealing at 523 K for 1 h, the total strain reduces markedly to ~4.1%, of which plastic strain is only 0.6%. In both the cases, high-resolution transmission electron microscopy and selected-area diffraction were utilized to confirm that no crystallization occurs during deformation. For the annealed film, *ρ* = 8.64 ± 0.05 g/cm^3^ whereas it is 8.5 ± 0.05 g/cm^3^ for the as-deposited film, showing a substantial reduction in the free volume content upon annealing. Furthermore, *ρ* of the as-deposited 50 nm thick film is lower than *ρ* values of 8.63 ± 0.08 and 8.76 ± 0.10 g/cm^3^ in 100 and 200 nm thick films respectively^65^, which indicate that the free volume content increases with a reduction in the film thickness. These preliminary experimental results demonstrate that an MG with a high free volume content and with a structure similar to that of a shear band, i.e., high and uniformly distributed free volume content, can exhibit extensive plastic deformation. Thus, present MD simulation study suggests that large homogeneous plasticity is possible in bulk MGs with large amount of free volume. This prediction deserves further experimental confirmation.

Concerning the comparison of results obtained from MD simulations with experiments, a few factors should be considered. In a laboratory experiment, it is difficult, if not impossible, to retain the SB structure exactly as that obtained in its plastically flowing state. In other words, the deformed sample (SB) would already experience much more relaxation than in MD high-rate flow state, as well as more post-SB relaxation while being brought to a subsequent tensile test. In such a case, the sample could be more like their MA state or 600 K for 5 ns relaxed state, rather than the fully rejuvenated SB state, and hence would again be a non-Newtonian flow regime and prone to strain localization. Furthermore, rapid cooling, such as 10^12^ to 10^13^ K/s, which is thought to be difficulty in laboratory at the moment, indeed can retain a highly rejuvenated structure. One point should be mentioned that the prediction by MD simulations has been recently supported by experiments^66^, e.g., using extreme high cooling rates, monoatomic metallic glasses can be indeed fabricated. Therefore, our MD results point out a direction for experiments to design such SB structure-like MG samples using novel techniques. In addition, recently various experimental validation for the size of the shear transformation zone (STZ)^67^,^68^,^69^,^70^,^71^,^72^,^73^, which is very relevant as nucleus for the shear band formation. From different measurements conducted on the nanoindentation, it is found that the STZ size are very relevant to the ductility of bulk metallic glasses^69^ and would be influenced by the structure state^70^, temperature and strain rate^71^,^72^,^73^. For the correlation between STZ size with its influence to shear band formation, more studies are still required.

## Conclusions

In summary, we have employed molecular dynamics simulations to perform a comprehensive investigation on the deformation behavior of Cu_64_Zr_36_, Cu_36_Zr_64_ and Ni_40_Zr_60_ metallic glass specimens, in which the atomic packing structure is akin to that in the shear bands. Homogeneous deformation was observed in all cases when the structure is ‘shear band’ like. The free volume content in these MG samples can be varied either by annealing, that reduces the free volume content in MGs, or by varying the cooling rate, that achieves different free volume contents in MGs. Results show that the deformation mode can change from homogeneous flow to localized flow depending on the free volume content in the MG sample. The observed deformation mode transition was correlated to the initial structure, which was analyzed first by recourse to the Voronoi polyhedra analysis and then with the structural open volume method. Following conclusions can be deduced from the present study.

1. Homogeneous deformation under tension was observed in Cu_64_Zr_36_ specimens with shear band like structures, and the response was observed to be size independent, suggesting that the shear band-like structure is one possible method to achieve tensile plasticity in metallic glasses with large size and monolithic amorphous structure. Studies on Cu_36_Zr_64_ and Ni_40_Zr_60_ yield similar results.
2. On the basis of both Voronoi polydedra and atoms near sub-atomic sized cavities, it was found that the plastic deformation can be better understood through some sort of threshold which enables the liquid-like regions to percolate through the MG. We further ascertain that such regions can be produced by processes such as rapid cooling, and then tuned by annealing.
3. MGs with relatively low density and shear band like structures can lead to the accommodation of large plastic deformation, which is useful in developing new MG alloys. *In-situ* tensile elongation experiments on thin Ni_60_Nb_40_ MG films reported in literature support this conclusion.

## Methods

MD simulations were first performed for the alloy Cu_64_Zr_36_ utilizing LAMMPS^74^, employing embedded-atom method potentials developed by Mendelev *et al.*^75^, with the following sequential steps: (1) ~10,000 atoms in a face-centered cubic (FCC) crystalline lattice cell were constructed. It was subjected to melting at 2000 K for 2 ns, which allowed for complete equilibrium, and then quenched to 50 K at a cooling rate of 10^11^ K/s in a NPT ensemble with a Nose-Hoover thermostat^76^. The final size of the sample is 5.6 (*x*)-5.6 (*y*)-5.6 (*z*) nm^3^; (2) A bigger sample with ~4,500,000 atoms and a size of 106.4 (*x*)-117.6 (*y*)-5.6 (*z*) nm^3^ was further constructed by replicating the sample generated in step (1) for 19 and 21 times in *x* and *y* directions, respectively. It was then relaxed at 50 K for 100 ps in the NPT ensemble to equilibrate the structure. This sample is referred as ‘BIG’ sample here afterwards; (3) The relaxed BIG sample was subjected to uniaxial tension along *y*-axis at 50 K with a constant strain rate of 1 × 10^8^ s^−1^ and a time step of 1 fs. Periodic boundary conditions (PBC) were applied in *y* and *z* directions whereas no constraint was imposed along *x*. The pressure in the *z* direction was adjusted to 0 kbar to allow for lateral contraction. The local von Mises strain^77^ of each atom, 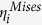, in the sample is computed by comparing the configurations of atoms before and after deformation. Von Mises strain is defined as 
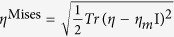
, where η and η_m_ are the local Lagrangian and hydrostatic strains for that atom, respectively. Atoms with 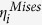 ≥ 0.2, were termed as ‘S-atoms’ and were utilized to visualize the plastic deformation within the specimen.

## Additional Information

**How to cite this article**: Zhong, C. *et al.* Deformation behavior of metallic glasses with shear band like atomic structure: a molecular dynamics study. *Sci. Rep.* **6**, 30935; doi: 10.1038/srep30935 (2016).

## Supplementary Material

Supplementary Information
