# Supplementary material for: Deformation behavior of metallic glasses with shear band like atomic structure: a molecular dynamics study
Source: Sci Rep. 2016 Aug 2;6:30935. doi: 10.1038/srep30935 (PMC4969605; doi:10.1038/srep30935)
Supplement: Supplementary Information [file srep30935-s1.doc]

**Supplement material**

**Deformation behavior of metallic glasses with shear band like atomic structure: a molecular dynamics study**

C. Zhong1, H. Zhang1,2, Q. P. Cao1, X. D. Wang1, D. X. Zhang3, U. Ramamurty1,4,5, and J. Z. Jiang1*

1*International Center for New-Structured Materials (ICNSM), Laboratory of New-Structured Materials, State Key Laboratory of Silicon Materials, and Department of Materials Science and Engineering, Zhejiang University, Hangzhou, 310027, People’s Republic of China*

*2Department of Chemical and Materials Engineering, University of Alberta, Edmonton, Alberta, T6G 2V4, Canada*

*3State Key Laboratory of Modern Optical Instrumentation, Zhejiang University, Hangzhou, 310027, People’s Republic of China*

*4Department of Materials Engineering, Indian Institute of Science, Bangalore-560 012, India*

*5Center of Excellence for Advanced Materials Research, King Abdulaziz University, Jeddah 21589, Saudi Arabia*

*) E-m*ail: jiangjz@zju.edu.cn*

**Figure captions**

Fig. S1 Tensile stress-strain responses of Cu_64_Zr_36_ SB samples at strain rate of 10^8^ /s up to a strain of 80%. Deformation maps at a tensile strain of 20%, 40%, 60% and 80% are shown in the inset.

Fig. S2 Tensile stress-strain responses of Cu_64_Zr_36_ SB samples at strain rate of 10^7^ /s, 10^8^ /s and 10^9^ /s. Deformation maps at a tensile strain of 20% are shown in the inset.

Fig. S3. Tensile SS curves of Cu_36_Zr_64_ (a) SB samples after annealing at different temperatures. SS curve obtained on SB and MA samples are also shown for comparison. Deformation maps (total strain = 20%) of samples annealed at 500 and 600 K, showing localization of flow in the latter. (b) Samples that were obtained with different cooling rates, ѱ. SS curves obtained on MA and SB samples are also shown for comparison. (b) Deformation maps (total strain = 20%) of MA samples subjected to ѱ = 10^11^ and 10^13^ K/s; flow localizes in the former whereas it is homogeneous in the latter. (c) Variations of Young’s modulus, *E*, and peak stress, σ_u_, of SB sample with the annealing temperature, T_a_ and samples with the cooling rate, *ψ*. (d) Variations of Poisson’s ratio, ν, and density, ρ, with T_a_ and *ψ.* Dashed lines in (c) indicate approximate T_a_ and ѱ, at which homogeneous to localized flow transition occurs.

Fig. S4. Tensile SS curves of Ni_40_Zr_60_ (a) SB samples after annealing at different temperatures. SS curves obtained on SB and MA samples are also shown for comparison. Deformation maps (total strain = 20%) of SB sample (annealed at 50K) and annealed at 800 K, showing localization of flow in the latter. (b) Samples that were obtained with different cooling rates, ѱ. SS curves obtained on MA and SB samples are also shown for comparison. (b) Deformation maps (total strain = 20%) of MA samples subjected to ѱ = 10^11^ and 10^15^ K/s; flow localizes in the former whereas it is homogeneous in the latter. (c) Variations of Young’s modulus, *E*, and peak stress, σ_u_, of SB sample with the annealing temperature, T_a_ and samples with the cooling rate, *ψ* . (d) Variations of Poisson’s ratio, ν, and density, ρ, with T_a_ and *ψ.*

Fig. S5. Variation of the fractions of Ni-centered Voronoi polyhedra (VP) in the SB sample of Ni_40_Zr_60_ with (a) the annealing temperature, T_a_, and (b) the samples with cooling rate, *ψ. *Zr-centered Voronoi polyhedra (VP) in the SB sample with (c) the annealing temperature, T_a_ and (d) with cooling rate, *ψ.* Only six most abundant VPs are considered.

Fig. S6. Variations of the fraction of atoms at the near-cavity region, ξ, in the SB sample with the annealing temperature, T_a_, and in samples cooled with different rates, *ψ,* in (a) Cu_36_Zr_64_ and (b) Ni_40_Zr_60_. Fractions of atoms at liquid-like region, ɸ, of SB samples with annealing temperatures, T_a_, and samples subjected to different cooling rates, *ψ,* in (c) Cu_36_Zr_64_ and (d) Ni_40_Zr_60_.

*
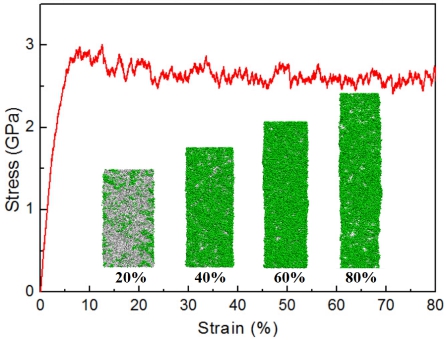
*

Fig. S1

**
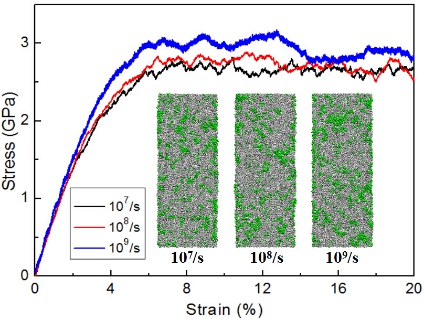
**

Fig. S2


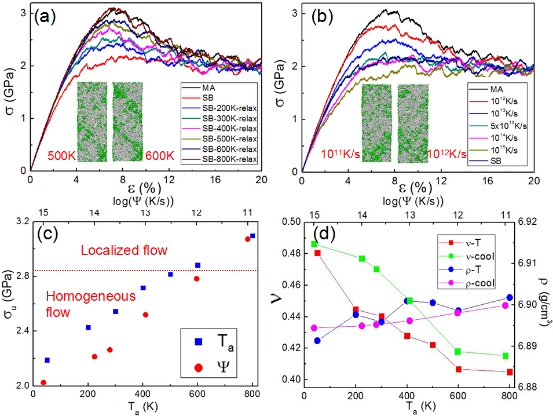


Fig. S3


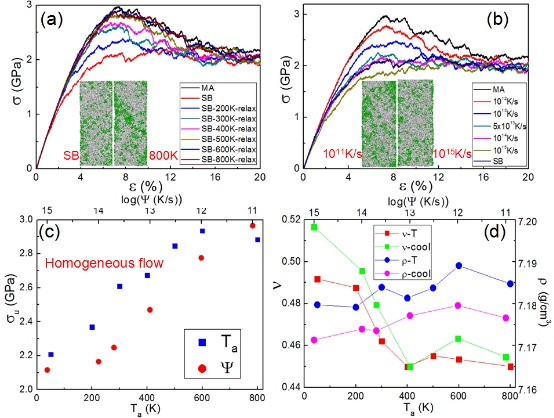


Fig. S4


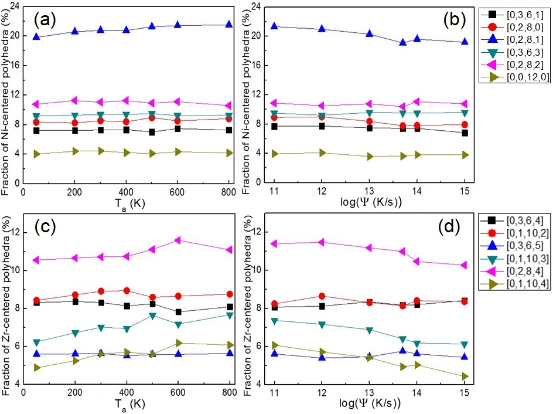


Fig. S5


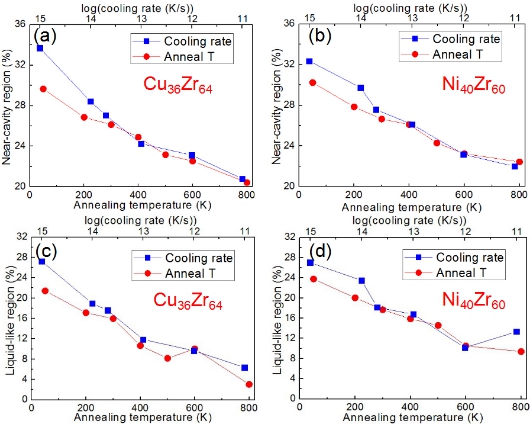


Fig. S6
